# Meckel’s Enterolith Causing Small Bowel Obstruction: A Useful Solution to a Unique Problem

**DOI:** 10.7759/cureus.15934

**Published:** 2021-06-26

**Authors:** Gabriel De la Cruz Ku, Erek Nelson, Rolando Calderon, Pouya Hemmati, Brian Kim

**Affiliations:** 1 General Surgery, Mayo Clinic, Rochester, USA; 2 Trauma and Acute Care Surgery, Mayo Clinic, Rochester, USA

**Keywords:** meckel´s diverticulum, gastrointestinal obstruction, surgery general, surgical acute abdomen, diagnosis

## Abstract

Meckel’s diverticulum (MD) is the most common congenital anomaly of the gastrointestinal tract. Its course is usually benign but may also result in complications requiring surgical intervention. A diverticulum may also permit the removal of intraluminal objects without bowel resection and anastomosis.

A woman in her 50s was found to have a mechanical small bowel obstruction secondary to an intraluminal mass within the terminal ileum. On exploration, an MD was encountered proximal to the mass. A diverticulectomy was performed after maneuvering the enterolith into the diverticulum.

Meckel’s diverticulum with an associated enterolith is a rare cause of small bowel obstruction. Historic imaging may show long-standing stones in the bowel lumen and provide a diagnostic clue. Diverticulectomy may be performed to reduce the risks of small bowel resection and anastomosis. This technique can be used for other intraluminal objects requiring removal in the presence of an MD.

## Introduction

Meckel’s diverticulum (MD) is caused by the incomplete obliteration of the vitelline duct during the seventh to eighth week of gestation [1]. It is present in approximately two percent of the population. Most MD are asymptomatic and, therefore, undiagnosed. A minority of those with MD will develop complications within their lifetimes. Diagnosis can be made by radiography, ultrasound, CT scan, or Technetium-99m pertechnetate. However, diagnosis is more challenging in adults compared to pediatric patients due to lower sensitivity and specificity [2]. An acute abdomen can be the initial presentation secondary to inflammation or bleeding. Less common presentations include herniation, malignancy, or obstruction. We present a case report and literature review of a small bowel obstruction caused by an enterolith formed within an MD. Although the presenting pathology is rare, benign intraluminal masses and MD are both more common. This case report demonstrates the technique of using an MD to extract an intraluminal mass.

## Case presentation

The patient is a woman in her 50s who presented to the emergency department (ED) with a two-day history of colicky abdominal pain in the mid-epigastrium and right lower quadrant associated with nausea, emesis, distention, and obstipation. She had a past medical history of hypertension and major depressive disorder treated with losartan and citalopram, respectively; and regarding her surgical history, she did not have any prior abdominal operation. She did not have symptoms or a history of biliary pathology.

On physical examination, her abdomen was distended and tympanitic, but nontender. Laboratory analysis revealed a normal white blood cell count and hemoglobin. Serum electrolytes were indicative of mild volume depletion. 

**Figure 1:**
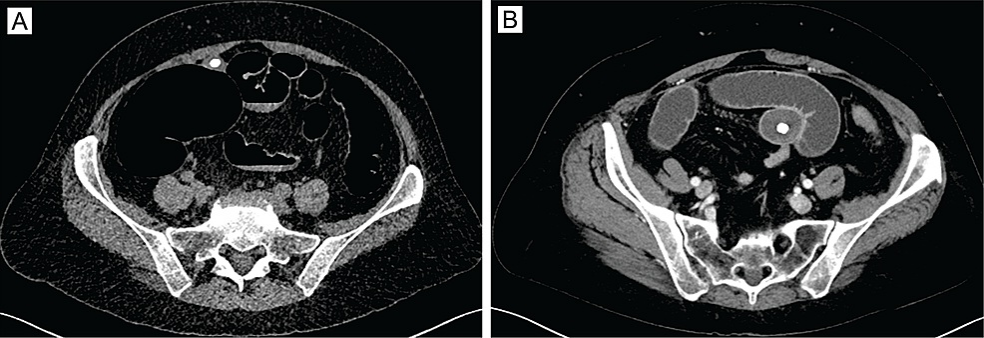
CT showing with Meckel's enterolith. (A) Historic imaging: radiopaque intraluminal mass was seen near the terminal ileum on a screening CT colonography performed six years prior to presentation. This mass was smaller than the mass that was removed. The lower-density periphery had grown in diameter, but the radiopaque core was identical in appearance. (B) Preoperative imaging: CT scan with IV contrast showing an obstructive mass with a calcified core in the terminal ileum. Small bowel is decompressed distal to the obstruction and dilated proximal to it.

She subsequently underwent a CT scan of the abdomen and pelvis which showed a distal small bowel obstruction with a transition point at a 2.9 cm round intraluminal structure with central calcification (Figure 1B). There was no evidence of small bowel ischemia or perforation. The patient did not have radiopaque cholelithiasis or imaging findings of cholecystoenteric fistula (Figure 1A,B). Upon review of prior imaging, screening CT colonography from six years prior for cancer screening purposes showed a similar, but smaller intraluminal structure associated with the terminal ileum (Figure 1A).

The differential diagnosis included teratoma or other benign or malignant mass, ingested foreign body, and gallstone ileus. While most surgeons hearing the history would suspect gallstone ileus, especially in patients with known biliary pathology, lack of visible cholecystoenteric fistula or inflammatory changes in the right upper quadrant precluded this diagnosis. The patient was a reliable historian and denied a history of unusual foreign body ingestion. While the imaging phenotype of a central calcification and concentric radiolucent material was reminiscent of a mature teratoma, this pathology arising in the small bowel of a middle-aged patient would be very unusual. Nevertheless, the mass had been present for at least six years and appeared to be enlarging. Therefore, an unknown malignancy was atop our differential diagnosis upon proceeding to the operating room.

**Figure 2:**
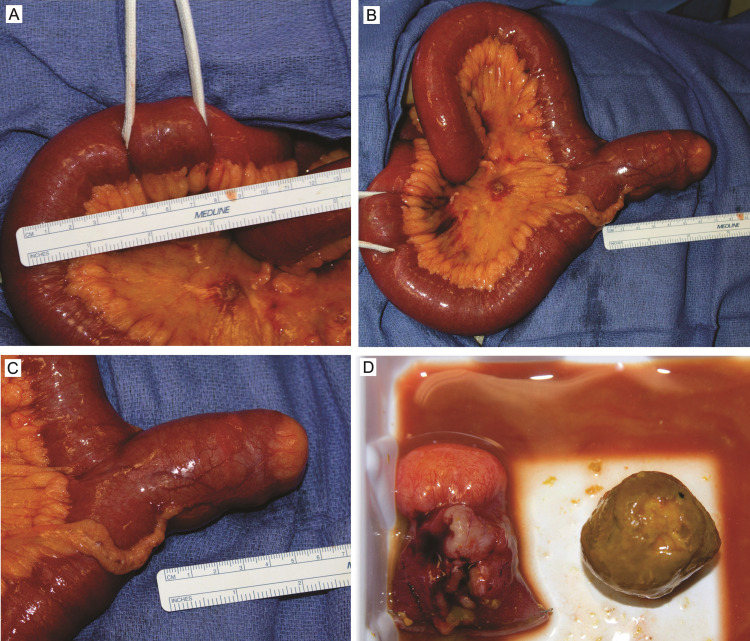
Intraoperative findings. (A) A three-centimeter, mobile, intraluminal, obstructive mass was discovered at the transition point from dilated to the compressed small bowel. (B and C) Proximal to this, a broad-based MD was identified that was seven centimeters in length. (D) The mass fit within the diverticulum and was confirmed to be an enterolith after diverticulectomy. MD, Meckel’s diverticulum

After nasogastric decompression, she was taken to the operating room for exploratory laparotomy via a limited periumbilical midline incision. Fifty centimeters proximal to the ileocecal valve, a broad-based MD seven centimeters in length was identified. Twenty centimeters distal to the MD, a mobile, intraluminal, obstructing enterolith was found. The stone was milked proximally into the lumen of the MD and it matched the diameter of the diverticulum (Figure 2A-D). A stapled, transverse diverticulectomy was performed with the stone within the diverticulum.

Postoperatively, the patient progressed without complication and was discharged on postoperative day five after the return of bowel function. Final pathology confirmed a benign MD without ectopic mucosa. The patient is currently alive and well and, as recurrence is unlikely status post diverticulectomy, she has been surgically dismissed.

## Discussion

Meckel’s diverticulum is present in approximately two percent of the general population and is the most prevalent congenital abnormality of the gastrointestinal tract [3-4]. Only 16% of patients with MD develop symptoms. Symptomatic MD is associated with age younger than 50 years, male sex, length greater than two centimeters, and presence of ectopic tissue such as gastric mucosa [5]. While the most common manifestation in children is hematochezia, obstructive symptoms are more common in adults [6-7]. Specifically, 36.5% of adults with symptomatic MD present with small bowel obstruction secondary to fixation of the free tip of the diverticulum to the anterior abdominal wall, 13.5% with intussusception, 12.5% with diverticulitis, 11.3% with hemorrhage, 7.3% with perforation, and 3.2% with malignancy [8]. Obstruction secondary to MD-associated enterolith is rare, and the preoperative diagnosis is challenging. Moreover, although laparoscopic MD resection is common, there are no reports of laparoscopic diverticulectomy with the removal of an obstructing enterolith [9-10].

After a systematic search of Pubmed, Embase, Google Scholar and Lilacs, we found 30 cases of MD enteroliths causing small bowel obstruction from 1959 to 2020 (Table 1) [11-39]. The median age at presentation was 58 years (interquartile range, IQR 42-72 years), with 73% of cases in males. Sixty-seven percent of cases were diagnosed with pre-operative imaging, although only 48% of enteroliths were radiopaque. Multiple stones were found in 23.5% of the cases. The average stone size was 3.6 cm (standard deviation, SD ± 1.03 cm), but ranged from 2.5 to 6 cm. Enteroliths were found more frequently in the terminal ileum (58%), while 38% remained within the MD.

**Table 1:** Previous case reports from small bowel obstructions caused by Meckel's enterolith. ND, no data; SB, small bowel; MD, Meckel’s diverticulum; CT, computed tomography; MRI, magnetic resonance imaging

No	Author	Year	Country	Age	Sex	Diagnosis	Imaging features	Size	Location of enterolith
1	Field et al. [11]	1959	USA	52	M	Surgical	Radiolucent	ND	SB
2	Danzis et al. [12]	1950	USA	ND	ND	ND	ND	ND	ND
3	Bergland et al. [13]	1963	USA	73	F	Surgical	Radiolucent	3cm	SB
4	Caridis et al. [14]	1965	France	ND	ND	ND	ND	ND	ND
5	Sbriccoli [15]	1969	Italy	ND	ND	ND	ND	ND	ND
6	Grosdidier et al. [16]	1972	France	ND	ND	ND	ND	ND	ND
7	Benhamou [17]	1979	France	ND	ND	ND	ND	ND	ND
8	Grant [18]	1981	Australia	65	M	Surgical	Radiolucent	ND	MD
9	Lopez and Welch [19]	1991	USA	85	M	Surgical	Radiopaque	3cm	MD
10	Rudge [20]	1992	USA	78	M	Surgical	Radiolucent	5cm	SB
11	McCallion et al. [21]	1992	Ireland	37	F	Surgical	Radiolucent	ND	SB
12	Kim et al. [22]	1999	Korea	58	F	X-ray/CT	Radiopaque	3 cm	SB
13	Tosato et al. [23]	2000	Italy	ND	M	Surgical	Radiolucent	ND	SB
14	Vasquez et al. [24]	2001	USA	60	M	X-ray/CT	Radiolucent	4cm	MD
15	Gamblin et al. [25]	2003	USA	24	M	Surgical	Radiolucent	ND	SB
16	Srinivas and Cullen [26]	2007	Belgium	84	F	X-ray	Radiopaque	Multiple	MD
17	Massoni Neto et al. [27]	2007	Brazil	43	M	X-ray	Radiopaque	Multiple	MD
18	Trésallet et al. [28]	2007	France	37	M	CT	Radiopaque	ND	SB
19	Rice et al. [29]	2010	USA	73	M	CT	Radiopaque	3cm	SB
20	Lai [30]	2010	Taiwan	9	M	Surgical	Radiolucent	ND	Cecum
21	Jones et al. [31]	2010	UK	56	M	X-ray/CT	Radiolucent	Multiple	MD
22	Gadhia et al. [32]	2010	India	55	M	X-ray/CT	Radiolucent	6cm	MD
23	Garrigós et al. [33]	2012	Spain	62	M	X-ray/US	Radiopaque	3cm	SB
24	Demetriou et al. [34]	2013	UK	30	F	X-ray/CT	Radiopaque	4cm	SB
25	Nguyen [35]	2014	Vietnam	60	F	X-ray/CT	Radiolucent	4cm	SB
26	Maurice et al. [36]	2016	Australia	72	M	X-ray/CT	Radiopaque	ND	SB and MD
27	Dill et al. [37]	2017	Australia	82	M	X-ray/CT	Radiopaque	Multiple	MD
28	Symeonidis et al. [10]	2017	Greece	48	M	X-ray/CT	Radiopaque	2.5cm	SB
29	Nastos et al. [38]	2017	Greece	16	M	X-ray/MRI/CT	Radiopaque	5cm	MD
30	Wauters et al. [39]	2018	Belgium	42	M	X-ray/CT	Radiolucent	ND	SB

The pathogenesis of these enteroliths is unclear but there are several plausible hypotheses. One theory is that the absence of ectopic gastric mucosa preserves an alkaline environment which, when combined with stasis of succus entericus due to the absence of peristalsis by the diverticulum, promotes precipitation of calcium salts and stone formation. Additionally, previous episodes of MD with associated local inflammation may promote stone formation [40-41]. We hypothesize that MD enteroliths resulting in obstruction are rare because an MD must be large and broad-based to be capable of harboring and expelling a stone of sufficient caliber to cause luminal obstruction. It is unlikely that a single stone is retained long enough in a broad-based MD for it to grow to such a size.

Like most bowel obstructions associated with an intraluminal mass, the definitive treatment is operative intervention. As in the case presented, diverticulectomy including the enterolith is the preferred option. However, if the stone cannot be returned to the MD, bowel resection including the diverticulum with primary anastomosis is safe and effective. Segmental small bowel resection and anastomosis are also recommended for MD with a base greater than one-third of the bowel circumference, presence of inflammation, ulceration or perforation at the base, or suspected small bowel ischemia [42].

Regarding the approach, we anticipated difficulties with visualization due to small bowel dilatation from mechanical obstruction and thus decided to undertake a primary open exploration. Due to the anatomic location of MD, any obstruction secondary to MD enterolith would result in dilation of the vast majority of the small bowel. Therefore, laparoscopic resection may prove difficult and perilous but may be successful in selected patients.

## Conclusions

In conclusion, despite MD is present in roughly 2% of the population and is usually asymptomatic, a wide variety of symptoms can develop at any age. All general surgeons will discover incidental MDs in their careers and resection of a normal-appearing diverticuli is not indicated but should be considered in the presence of observed or suspected pathology including enterolith. Moreover, when performing abdominal exploration to extract a mobile intraluminal body such as an enterolith, obstructing gallstone, or ingested object, an MD can serve as a useful vehicle for extraction while avoiding bowel anastomosis.
